# Food safety news events classification via a hierarchical transformer model

**DOI:** 10.1016/j.heliyon.2023.e17806

**Published:** 2023-06-30

**Authors:** Shufeng Xiong, Wenjie Tian, Vishwash Batra, Xiaobo Fan, Lei Xi, Hebing Liu, Liangliang Liu

**Affiliations:** a Henan Agricultural University, Zhengzhou, 450002, China; b School of Computer Science and Mathematics, Keele University, ST5 5AA, Keele, UK

**Keywords:** Food safety, BERT, Transformer, Deep learning, Multi-classification, Natural language processing

## Abstract

In light of the significance of regulatory authorities and the rising demand for information disclosure, a vast amount of information on food safety news reports is readily accessible on the Internet. The extraction of such information for precise classification and provision of appropriate safety alerts based on their respective categories has emerged as a challenging problem for academic research. Given that most food safety-related events in news reports comprise lengthy text, the pre-trained language models currently employed for text analysis are generally limited in their capability to handle long documents. This paper proposes a long-text classification model utilising hierarchical Transformers. We categorise information in long documents into two distinct types: (1) multiple text chunks meeting the length constraint and (2) essential sentences within long documents, such as headings, paragraph start and end sentences, etc. Initially, our proposed model utilises the text chunks as input to the BERT model. Then, it concatenates the output of the BERT model with the important sentences from the document and use them as input to the Transformer model for feature transformation. Finally, we utilise a classifier for food safety news classification. We conducted several comparative experiments with various commonly used text classification models on a dataset constructed from publicly available information on food regulatory websites. Our proposed method outperforms existing methods, establishing itself as the leading approach in terms of performance.

## 1 Introduction

Food safety is a crucial issue for national development and people's lives. Information technology's rapid development has led to many new research directions in ensuring food safety. For instance, using information retrieval techniques for obtaining important information about food safety. Furthermore, the analysis of this data holds immense importance in addressing numerous food safety issues. The rising scrutiny and attention of regulatory authorities and their demand for information disclosure has also led to more food-related information being readily available publicly, including food safety-related news, reviews, etc [1]. In the face of tens of thousands of food safety information pieces generated daily, how to accurately classify and provide corresponding safety warnings [2], [3] as per its category has also emerged as a critical problem that needs attention. The information obtained after classification can be useful in food recalls [4], analysis and detection of food safety issues in online food ordering through review text mining [5], etc.

As previously described, the classification of food safety events is an essential step in the food safety warning process [6]. However, the first step in text analysis is often to classify information sources after obtaining them by manual or coarse classification based on keywords automatically. Although there are various direct studies on the extraction of entity relationships within the domain of food safety [7], [8], the construction of knowledge graphs of food safety events and questions and answers [9], and the classification of topics in food safety publications in the knowledge base [10]. However, such coarse classification of the text is detrimental to not only the correctness of the classification stage but also the correctness of the results of other subsequent stages. Therefore, this paper defines the following four categories of news related to food safety events crawled on the Internet: additives, heavy metals, dairy products, and counterfeits, respectively.

Our task is essentially a text classification problem, which is a significant and applicable research focus within the field of NLP (Natural Language Processing) [11]. The specific manifestations of text classification include Sentiment Analysis [12], Question Answering [13], Relation Extraction [14], NER (Named Entity Recognition) [15] and so on. The current popular neural text classification algorithms rely on neural language models, of which BERT (Bidirectional Encoder Representations for Transformers) [16] is a typical representative. It highlights the adoption of a novel approach called MLM (Masked Language Modeling) rather than conventional one-way language models or shallow combination of two one-way language models for pre-training. This enables the generation of deep bidirectional language representations. However, it should be noted that BERT is limited to input sequences with a maximum length of 512 tokens. It is a significant limitation because many standard document types are much longer than 512 words. Moreover, most texts on food safety events exceed this limit, thus making the long text analysis less accurate. The experiments in this paper also verify that BERT is inefficient for long text classification processing.

To address the aforementioned challenges, this paper presents a hierarchical architecture scheme that combines BERT and Transformer. This scheme is designed specifically to tackle the issue of long text classification in the context of food safety events. Specifically, our model divides the text of food safety news events into segments of 512 characters for cutting and uses each segment as a separate BERT input. Finally, the BERT output of each segment, the headline, and the first and the last sentence of the safety news event are combined as new input for the Transformer model for feature learning. The learned features are subsequently fed into the classifier for classification prediction. The model makes full use of the features of BERT to transform from long text to short text, obtain the semantic representation of text fragments, document title, and first and last sentences of paragraphs by BERT, and then use Transformer to further construct the document feature representation before classification, which helps in achieving more accurate text classification results.

In this paper, we propose an improved idea of hierarchical Transformers to remedy the problem of long text classification of food safety events. The novelty of the proposed model stems from the integration of BERT and Transformer into a hierarchical architecture designed specifically for long text classification. Our model segments text and extracts features using models at various levels, thereby capturing both local and global information - resulting in enhanced accuracy. Furthermore, it combines multiple input sources such as titles, first/last sentences, to enrich the text representation and improve overall performance. By leveraging BERT's ability to convert long text into shorter representations and capture semantic meaning, the model can better understand contextual nuances and improve classification accuracy. Additionally, Transformer facilitates feature learning and document representation, enabling robust modeling and parallel processing for extracting significant features, further enhancing the performance. To verify the effectiveness of the proposed approach, we conducted several experimental studies on real-world news data of food safety events and compared our model with commonly used text classification models such as BERT, FastText, TextCNN, etc.

The primary contributions of this paper can be summarized as follows.

• we focus on the first step in food safety monitoring events and use NLP techniques to classify text, improving the classification efficiency and thus saving a lot of human labour and cost.
• we propose a new hierarchical transformer text classification algorithm to classify long documents in food safety events.
• We have completed experimental validation on a real dataset, and our model achieves the current state-of-the-art performance.

The structure of the paper is outlined as follows: In Section 2, an introduction is provided to highlight relevant prior research. Section 3 elaborates on the key components of the model proposed in this paper. The experimental results of the proposed model, compared with other models, are presented in Section 4 to assess its performance. Section 5 includes a discussion on the methodology employed in this paper and explores potential future research directions. Finally, Section 6 provides a summary of the main findings of the paper.

## 2 Related work

Food safety is a topic of great public concern, and analyzing news events related to food safety is crucial for gathering information and data in this field [17], [18]. NLP, a branch of artificial intelligence, can automate the extraction of implicit information from text, providing technical support for the analysis of food safety news events. By utilizing NLP techniques, critical information can be identified in the text, enabling food safety authorities to quickly respond and take effective measures [19], [18]. Regarding the analysis of food safety news event texts, Xiao et al. [20] proposed a method for detecting food safety events using multi-feature fusion. They introduced a news representation approach that combines TF-IDF features, named entity features, and headline features. However, their feature selection and weight setting methods have subjective elements that require further optimization and validation. Zuo et al. [21] developed a Two-Dimensional Semantic Classification Model of Feature Words called CNN-PABLSTM to identify internet food safety violations. While their method achieved satisfactory results, larger datasets and more complex network structures are needed to further enhance the classification performance. Wang et al. [22] constructed a BERT-BiLSTM (Bidirectional Long Short-Term Memory) model for food opinion-based entity relationship extraction and conducted comparative experiments on a food sentiment dataset. However, these methods limit the classification performance of the model due to deficiencies in the semantic representation of the underlying model text, although the corpus characteristics of the food safety event domain are taken into account.

Text classification is a crucial and practical research field in NLP. Over the past decade, text classification has transitioned from shallow learning models to deep learning models [23]. Deep learning techniques utilize data to autonomously acquire text features, eliminating the need for traditional manual feature selection methods [24]. This shift towards general learning processes for feature selection eliminates subjectivity and randomness associated with manual feature selection. Kim [25] introduced the use of CNN (Convolutional Neural Network) in text classification tasks, simplifying text extraction and enhancing classification accuracy. Another widely adopted deep learning model for text classification is the RNN (Recurrent Neural Network) framework [26]. RNN have demonstrated their effectiveness in learning contextual information from text, leading to excellent classification performance. However, traditional RNN suffer from the issues of vanishing and exploding gradients as the input volume increases. To address the limitations of traditional RNN models, Hochreiter et al. [27] developed the LSTM (Long Short-Term Memory) model by incorporating gate mechanisms. LSTMs have proven effective in overcoming the gradient vanishing and exploding problems. Consequently, LSTM has been widely applied to text classification tasks, yielding remarkable results [28]. These general text classification methods have some shortcomings in the representation of domain features.

In recent years, attention mechanisms have widely gained popularity in the field of NLP, which is dominated by deep learning-based models. Bahdanau et al. [29] were pioneers in integrating the attention mechanism into recurrent neural networks (RNNs) for machine translation tasks. Yin et al. [30] introduced a hybrid approach combining convolutional neural networks (CNN) and attention, whereas Zhou et al. [28] applied the attention mechanism to Bidirectional Long Short-Term Memory (BiLSTM) models in the context of text classification. Their objective was to extract essential semantic information from sentences. Despite these advancements, certain aspects, such as the internal location semantics, still pose challenges in terms of learning and processing. The introduction of BERT by Devlin et al. [16] marked a significant milestone in the development of text classification and other NLP techniques. BERT generates contextual word vectors and has demonstrated superior performance across various NLP tasks, including text classification. However, BERT's self-attention mechanism has limitations when dealing with long texts, which is a common characteristic of inputs in the field of food safety news events.

This paper's work on food safety news event classification using NLP techniques belongs to the field of food safety and risk analysis. By using the latest NLP techniques, we aim to address a research gap in the automation and effective classification of food safety news events, which belongs to a domain-specific long text classification problem. The application of BERT and Transformer models allows us to capture complex semantic relationships and contextual information to improve the performance of event classification.

Our approach is a fusion of the powerful semantic representation capabilities of BERT and Transformer. By segmenting long texts, we can obtain more precise semantic information for each document. The output is then combined with important sources of information in the document, such as the headline and the first and last sentences of the document. This combined representation is then fed into the Transformer model to perform the feature representation. Finally, this representation is connected to the classification layer to make predictions.

## 3 Methods

This paper employs a new fusion model combining BERT and Transformer for classification prediction to enhance the accuracy of long text classification for food safety news events. Specifically, it converts lengthy texts into shorter ones to serve as input for BERT, enabling the extraction of detailed feature information through BERT's training. Subsequently, the Transformer model is trained to refine the text classification results and enhance their precision. Specifically, we represent a document in chunks as a set D={i=1,...,N} consisting of N blocks of documents, N indicates the number of chunks of the document after splitting, the specific value being the actual number of chunks after the entire document has been split into segments of 500 characters in length. Each document block consists of a BERT input by adding the first and last [CLS] and [SEP], and the mapping is obtained through the output: $E_{1}⟶h_{1}$. Each document block corresponds to an output result, and the output results of all document blocks plus the first and last sentences of the document and the key words in the sentence form a new set $T=\left\{c_{1},\cdots ,c_{n},W_{1},\cdots ,W_{n}\right\}$. We take the set T as the input to the Transformer and get the final output through the Transformer's attention mechanism. By the final classification result, we can get the final class label as 1, 2, 3, 4 to determine which category he belongs to.

**Figure 1:**
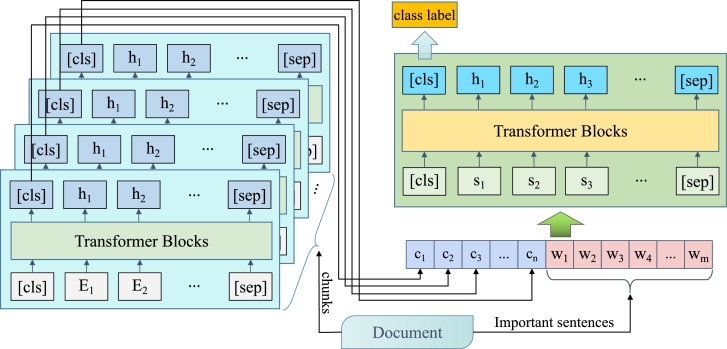
Our model: Hierarchical Transformer.

The model diagram presented in this paper comprises three modules: the document chunking module, the feature integration module, and the final classification module. The document chunking module employs a variable number of BERT models, which are primarily responsible for segmenting text documents based on specific rules. The feature integration module primarily utilises Transformer models, which integrate the features obtained from document chunking along with important sentences such as the title, first and last sentence of each document. The classification module's primary role is to perform text classification based on the output obtained from the integration module. The Fig. 1 illustrates the model architecture.

### 3.1 Document chunking module

Pre-trained language models are limited in their ability to process input text due to performance and generality considerations. For example, BERT can usually only handle text up to 512 characters in length. The model does not process text exceeding this limit, resulting in lost information. To address this issue, a possible solution is to split long texts into multiple shorter ones that meet the length constraint. Each short text can then be encoded independently using a separate BERT model, fully utilising the pre-training capability to capture local semantic and focused information. This approach can provide richer information for subsequent document integration and classification modules, resulting in an improved overall classification performance.

Text data is typically unstructured and cannot be directly input into models. Instead, the BERT model requires word vectors for each word in the text as its main input. These vectors can be initialised randomly or using an input vector such as Word2Vector [31] as the initial value. The output is a vector representation of each word that incorporates the full semantic information of the text. Due to limitations within the BERT module, the length of each text sequence must be 500 characters or less. To preprocess the input data, a special marker [CLS] is added at the beginning of each text sequence to indicate whether it is a standalone text or part of a sentence pair. Additionally, [SEP] markers are used to indicate the separation between sentences. To divide text with length D into segments of length k, we can use the following Eq. (1) to create m groups of text segments.

(1) m=split(D,k)

The BERT model takes in a set of sentences as its initial input. By accessing the word vector table, every word within the text is transformed into a one-dimensional vector, which acts as the input for the model. The model's output is a fused vector representation that captures the comprehensive semantic information of each word in the input text, as shown in Eqs (2) through (3).

(2) $$\text{E}=\sum_{i=1}^{n}E_{i}$$

(3) $${\text{h}}_{1}=BERT(E_{1})$$

where $h_{1}$ denotes the vector representation after fusing the full-text semantic information. In our task, $E_{i}$ refers to the text section of the *i*th news item. The BERT input vector comprises three vectors: a word embedding, a segment embedding, and a position encoding. The word embedding represents each word using a vector derived from BERT. BERT is trained on sentence pairs and employs segment embedding vectors to distinguish between different sentences. The positional encoding vector assists BERT in recognizing the positional information of each word through learned positional encoding techniques.

During data processing, BERT processes each text block independently, allowing multiple text blocks to be processed in parallel and increasing overall processing speed.

### 3.2 Document integration module

When text is processed by the document chunking module, it is divided into several text chunks. However, the processing results of each chunk are independent of each other and can lack global contextual information. In order to fully comprehend the information presented in a text and extract important details, it is essential to consider the context and background provided within the text. This context is typically broad in scope and encompasses a global viewpoint. The document integration module exists to integrate information from individual text blocks, resulting in a global text representation.

To achieve this, the document integration module uses the Transformer [32] model with an attention mechanism to model relationships between different text blocks. The Transformer Encoder model takes the word embedding representation of a sentence and its corresponding location encoding information as input. The multi-headed attention mechanism at the model's core performs separate computations using multiple attention mechanisms to obtain more levels of semantic information. The results obtained by each attention mechanism are then stitched together to obtain the final result. Finally, the Transformer converts the output of the multiple attention layers into the final output through feed-forward fully connected layers. Each layer transforms the data, adds nonlinearity through an activation function, and outputs the final word vector matrix, as shown in Eqs (4) through (5).

(4) $$\text{I}nput=\left\{c_{1},\cdots ,c_{n},W_{1},\cdots ,W_{n}\right\}$$

(5) Output=TB(Input)

Where $c_{i}$ represents the output of each BERT, $W_{i}$ is the word embedding of each word int important sentences such as news headlines, first and last sentences in each paragraph and *TB* is the core block of Transformer.

The activation function of our module is the ReLU (Rectified Linear Unit) function, which is a nonlinear activation function. It can effectively enhance the nonlinear capability of the model and make the model fit complex data better. As shown in Eq. (6).

(6) f(x)=max⁡(0,x)

where f(x) denotes the output value, *x* denotes the input value, and max denotes taking the maximum value.

### 3.3 The classification module

The document chunking module and the document integration module process documents to obtain a global text representation that contains the semantic information of the entire text collection. The objective of document classification is to map this global text representation into predetermined categories, achieving the aim of text classification. After the fused features are processed by the Transformer network and the fully connected layer, the final classification results are obtained. The loss function is defined using binary cross-entropy, as shown in Eq. (7).

(7) $$\text{L}=-\sum_{i=1}^{n}y_{i}\log p({\overset{ˆ}{y}}_{i}|x_{i})$$

where *y* denotes the true category, $\overset{ˆ}{y}$ denotes the predicted category, p(y|x) denotes the probability of model prediction, and *n* denotes the number of training samples.

## 4 Experiment

### 4.1 Data preparation

We obtained our experimental data from several popular news websites in China that provide valuable real-time information. The performance and robustness of our approach in the food safety domain were assessed using the provided news data. We named the dataset “Food Safety News Events” and manually labelled the data. In order to keep the authenticity of the labeled data, the same data is labeled several times independently and compared among the labeled personnel. By comparing the tagging results, the consistency and accuracy between taggers is checked. According to the Chinese Food Safety Law, food safety incidents can be classified into nine categories. Based on the analysis of the original corpus collected, four categories of typical food safety events were finally manually labelled. The specific category definition information is as follows: Class1 for fake and shoddy products, Class2 for food safety incidents of additives, Class3 for pesticide residues and heavy metals, and Class4 for dairy products. The datasets were divided into development, train, and test sets using a ratio of 1:8:1. The statistics for each set are presented in Table 1.

**Table 1:** The statistics of the dataset.

	Amount	Class1	Class2	Class3	Class4
dev	416	206	23	170	17
train	3327	1646	181	1364	136
test	413	205	22	170	16

### 4.2 Evaluation metrics

In this study, we evaluate the performance of the model using Accuracy, Precision, Recall, and F1 score as metrics. Accuracy measures the proportion of correctly predicted labels. Precision represents the ratio of correctly identified labels to all identified labels by the model. Recall is the ratio of true results to all correctly predicted results. The F1 score is a weighted average of precision and recall, providing a comprehensive evaluation of the model's performance. The evaluation metrics are defined by the following Eqs (8)-(11).

(8) $$\text{Accuracy}=\frac{TP+TN}{TN+TP+FN+FP}$$

(9) $$\text{Precision}=\frac{TP}{\left(FP+TP\right)}$$

(10) $$\text{Recall}=\frac{TP}{\left(FN+TP\right)}$$

(11) $$\text{F1}=\frac{2\times \text{Precision}\times \text{Recall}}{\left(\text{Precision}+\text{Recall}\right)}$$

In the evaluation metrics, TP denotes the count of positive classes correctly predicted as positive, FN (False Negatives) represents the count of positive classes predicted as negative, FP (False Positives) signifies the count of negative classes predicted as positive, and TN (True Negatives) indicates the count of negative classes correctly predicted as negative. The sum of these four values provides a total number of samples tested.

Moreover, since this paper involves multi-classification, we also use macro avg and weighted avg as evaluation metrics. The formulas for the evaluation metrics are shown in Eqs (12)-(14).

(12) $$\text{Macro Precesion}=\frac{1}{n}\sum_{i=1}^{n}P_{i}$$

(13) $$\text{Macro Recall}=\frac{1}{n}\sum_{i=1}^{n}R_{i}$$

(14) $$\text{Macro F1}=\frac{1}{n}\sum_{i=1}^{n}F1_{i}$$

### 4.3 Experiment settings

The parameters of the basic text encoding model, specifically the BERT-Base model, are frozen. To achieve optimal performance, the batch size for input sequences is set to 16, the text modal has a hidden layer dimension of 32, and the limit for the maximum input sentence length is set at 512. To prevent overfitting, a layer normalization layer is added after each fully connected layer, and a dropout layer with a rate of 0.6 is incorporated. The Adam optimization method is utilized, with an initial learning rate of 1e-3, and the maximum training period is set to 100.

### 4.4 Baselines

To evaluate the efficiency of our proposed model, we performed a comparative analysis by comparing its performance with several commonly used models. Our objective was to assess the performance and robustness of our proposed model by comparing it with other models. We selected various popular text classification models and tested them on the same dataset. Subsequently, we evaluated the performance of various models by analyzing their Accuracy, Precision, Recall, and F1 scores.

• **BERT**: The model takes a raw word vector of individual words in the text as input, which can be randomly initialised or initialised using a pre-training algorithm like Word2Vec. It outputs a word vector representation of the text incorporating full semantic information. By probing the contextual relationships between words in the input text and identifying different meanings of the same words in different sentences, it enhances the probability of text classification [16].
• **BERT-CNN**: This model combines the features of both BERT and CNN algorithms. BERT probes word contextual relationships and different meanings in different sentences, while CNN further extracts local semantic features of text vectors to enhance the text classification probability.
• **BERT-RNN**: This model is similar to the aforementioned BERT-CNN, but the difference lies in replacing CNN with RNN. The purpose of the RNN module is to capture text semantic information over longer distances.
• **FastText**: The model takes a raw word vector of individual words in the text as input, which is randomly initialised. Multiple words represented by vectors are then input, and specific target values are output. The hidden layer is created by taking the average of multiple word vectors and superimposing them. The next step is to construct the fully connected layer and then generate the classification probabilities by softmax layer. [33].
• **TextCNN**: The model pads all sentences to the same length, performs loading of pre-trained word vectors or random initialisation, and computes the input via convolution in the convolution layer before entering the pooling layer. Based on the output of the pooling layer and the number of categories, a fully-connected layer is built. The final classification result is obtained by applying softmax [25].

### 4.5 Experimental results

To validate the efficacy of our method, we performed a comparative analysis with multiple baseline models. We used the weighted average of the four classifications as a measure, and the results are shown in Table 2.

**Table 2:** Classification results of existing and our model.

	Accuracy	Precision	Recall	F1
BERT	0.7627	0.7645	0.7627	0.7589
BERT-RNN	0.7990	0.8147	0.799	0.7983
BERT-CNN	0.7748	0.8037	0.7748	0.778
FastText	0.8208	0.8206	0.8208	0.8136
TextCNN	0.8087	0.8076	0.8087	0.8071
**Our Model**	**0.8402**	**0.8372**	**0.8402**	**0.8349**

The performance of our model was assessed by comparing it with other methods on a test dataset. The results demonstrated that our model outperformed all other methods in terms of these metrics, achieving percentages of 84.02, 83.72, 84.02, and 83.49, respectively.

Comparatively, FastText achieved 82.08, 82.06, 82.08, and 81.36 accuracy, precision, recall, and F1 values, respectively, while BERT-RNN and BERT-CNN obtained 81.47 and 80.37 percentages for precision, respectively. Finally, TextCNN achieved percentages of 80.87, 80.76, 80.87, and 80.71 for accuracy, precision, recall, and F1, respectively. However, BERT-RNN and BERT-CNN were found to be less effective than these models in other metrics. The findings clearly demonstrate that our model exhibits superior performance when compared to other methods. Additionally, we evaluated the performance of the original BERT for our task. We truncate the long text directly (retaining the first 512 words) as input to the BERT model, leaving the rest of the model unchanged. The BERT model achieved percentages of 76.27, 76.45, 76.27, and 75.89 for accuracy, precision, recall, and F1, respectively. This further demonstrates the superiority of our model in long text classification.

To account for differences in the number of samples per class, macro average was calculated for each method, and the results are presented in Table 3.

**Table 3:** Macro avg of existing and our model.

	Precision	Recall	F1
BERT	0.7931	0.6325	0.6888
BERT-RNN	0.6783	0.7635	0.7059
BERT-CNN	0.6722	0.7365	0.6880
FastText	0.7855	0.6904	0.7126
TextCNN	0.7161	0.6786	0.6951
**Our Model**	**0.7970**	**0.721**	**0.7487**

**Figure 2:**
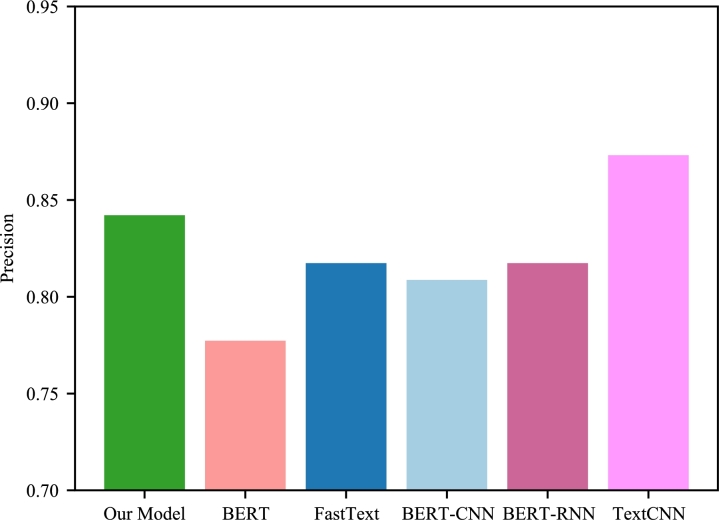
Class1 in the respective models Precision.

**Figure 3:**
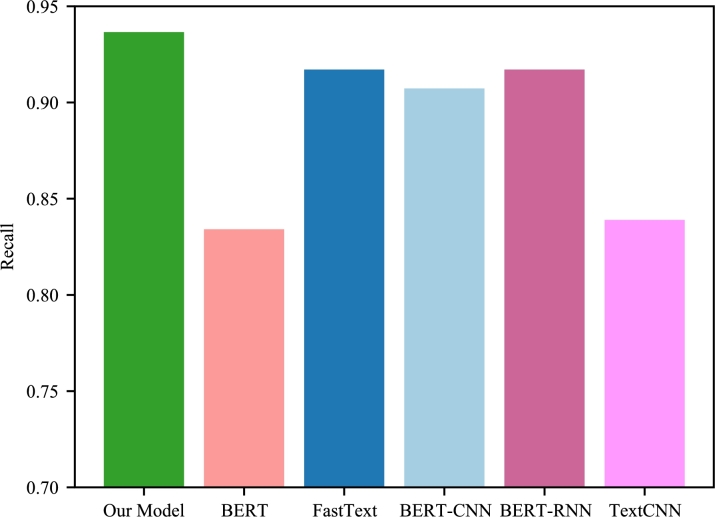
Class1 in the respective models Recall.

**Figure 4:**
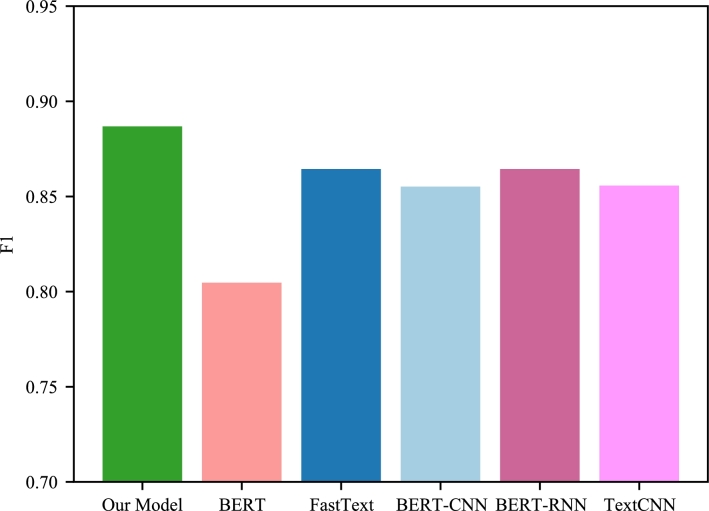
Class1 in the respective models F1.

In addition to demonstrating exemplary overall performance, our model exhibits notable advantages in diverse classifications. To elaborate further, we present a comparison of various models under precision, recall, and F1 metrics for Class1 as an illustrative example. As can be observed in Figure 2, Figure 3, Figure 4, our model outperforms its counterparts in Class1, which serves as compelling evidence of its efficacy.

In order to observe in a more detailed way the differences in the models on the different categories of classification, we also statistically evaluated the results on each category, as shown in Table 4.

**Table 4:** Results of the four classifications on different models.

	Classification	Precision	Recall	F1
BERT	Class1	0.7773	0.8341	0.8047
	Class2	0.7692	0.4545	0.5714
	Class3	0.7368	0.7412	0.7390
	Class4	0.8889	0.5000	0.6400

BERT-RNN	Class1	0.8174	0.9171	0.8644
	Class2	0.3704	0.4545	0.4082
	Class3	0.8855	0.6824	0.7708
	Class4	0.6400	1.0000	0.7805

BERT-CNN	Class1	0.8087	0.9073	0.8552
	Class2	0.3250	0.5909	0.4194
	Class3	0.8710	0.6353	0.7347
	Class4	0.6842	0.8125	0.7429

FastText	Class1	0.8174	0.9171	0.8644
	Class2	0.7778	0.3182	0.4516
	Class3	0.8408	0.7765	0.8073
	Class4	0.7059	0.7500	0.7273

TextCNN	Class1	0.8731	0.8390	0.8557
	Class2	0.5000	0.4091	0.4500
	Class3	0.7772	0.8412	0.8079
	Class4	0.7143	0.6250	0.6667

Our Model	Class1	0.8421	0.9366	0.8868
	Class2	0.6923	0.4091	0.5143
	Class3	0.8535	0.7882	0.8196
	Class4	0.8000	0.7500	0.7742

According to the Table 4, it can be observed that the performance on Class1 and Class3 generally better than Class2 and Class4 in four specific classification metrics, especially on Class1. Our model outperforms the other models in terms of classification results for both Class1 and Class3. Additionally, the performance of our model in Class2 and Class4 is comparable to that of the other methods. In addition, other approaches also perform well in different classification metrics, for example, TextCNN achieves a percentage of 87.31 in precision on Class1. BERT-RNN, BERT-CNN and FastText also perform well in recall on Class1 and other classification metrics.

According to the experimental findings, it is evident that our proposed model surpasses the performance of the current baseline models.

### 4.6 Error analysis

**Figure 5:**
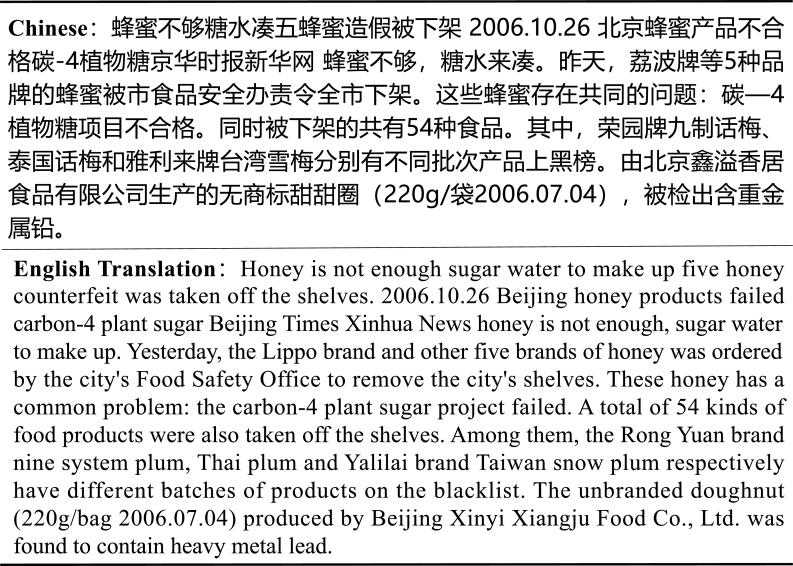
Example of a model predicting incorrect text input.

In our model, our task is to perform a classification task using the BERT and Transformer models with the goal of classifying the input sentences correctly. However, the model is also subject to some typical errors. We will provide a concrete example to analyze the causes of model errors and possible directions for improvement. There is a document that the contents are in Fig. 5.

It is categorised as Class3, but should be Class2. Our model over-focused on the last sentence. Although this sentence refers to “heavy metal lead”, it does so in the context of describing the detected food product, “unbranded doughnuts”. This sentence does not correlate well with the overall description of the problematic honey incident in the document; it is only a pass-by mention of a sample of the same batch in the document. The model does not understand this contextual relationship well and therefore incorrectly identifies the entire document as Class2. In this example, the entire text is filled with information about honey, from the headline to most of the content. However, because the model alone gives a high representation weight to each word in the last sentence of the document (although this improves the overall performance), it leads the model to make incorrect classifications on individual samples. This suggests that we are able to introduce a global attention mechanism in which the representation weights of different parts of the text are adjusted based on an understanding of the overall semantic information of the document, to ensure effective semantic representation of the model at the document level.

## 5 Discussion

We introduce a novel method for classifying long text food safety news using BERT and Transformer models. The experimental results reveal that our method achieved diverse outcomes on four different classifications due to the discrepancy in data samples. Specifically, better results were obtained on Class1 and Class3 due to the larger data samples of them. However, the classification results of BERT-RNN, BERT-CNN, and BERT models were significantly inferior to those of TextCNN and FastText models on different models due to the limitations of the BERT model. This finding provides further evidence of the limitations of BERT in handling long text classification tasks. Nonetheless, our model overcomes the shortcomings of BERT itself and achieves good results in long text processing. The experimental results provide compelling evidence of our model's exceptional feature extraction and classification performance, making it a more practical choice compared to other neural network models.

Nevertheless, it needs to be acknowledged that our approach also has certain unavoidable limitations. While pretrained models like BERT offer general language understanding, there is a need for domain-specific pretraining to capture the nuances and terminology specific to food safety. Therefore, we will explore domain adaptation techniques to enhance the performance of the method on food safety news data. Because the data used in this study are derived from past events, guaranteeing real-time event detection is not possible. In the future, we plan to merge multiple data sources to develop a general event detection method for rapid event classification detection. Additionally, some classifications lacked sufficient data in this study. Therefore, we intend to gather more actual data and expand the samples of different categories to improve our model's training.

## 6 Conclusions

In this paper, we propose a hierarchical Transformer model for classifying long text food safety news. To address the challenge of processing long text data, we adopt a document chunking approach that partitions long text into multiple document chunks to enable better processing. Each document chunk is treated as a separate input to the BERT model for preprocessing, which automatically extracts important features from the text, thereby increasing the semantic informativeness of the text representation. The BERT output of each document block, along with the first and last sentences of the document, serve as input to the Transformer that utilises an attention mechanism to capture correlation information in the text and gain a deeper understanding of its semantics. This enables us to better comprehend long text data and classify it effectively. Compared to traditional methods, our approach does not require advanced annotation, thus reducing the workload of data annotation. Additionally, we present a novel long text classification model algorithm that can better handle long text data and enhance the effectiveness of classifying long food safety news event texts. This is vital for further research on food safety issues, improving government reputation, and reducing social anxiety and economic loss. Our proposed method has significant implications for practical applications in the field of food safety. By accurately classifying long text news events related to food safety, we can promptly respond to food safety issues and take measures to mitigate potential risks, thereby safeguarding public health and maintaining social stability.

## CRediT authorship contribution statement

**Shufeng Xiong:** Conceived and designed the experiments; Performed the experiments; Wrote the paper. **Wenjie Tian:** Contributed reagents, materials, analysis tools or data; Wrote the paper. **Vishwash Batra:** Analyzed and interpreted the data; Wrote the paper. **Xiaobo Fan:** Contributed reagents, materials, analysis tools or data. **Lei Xi:** Conceived and designed the experiments. **Hebing Liu:** Analyzed and interpreted the data. **Liangliang Liu:** Conceived and designed the experiments.

## Declaration of Competing Interest

The authors declare that they have no known competing financial interests or personal relationships that could have appeared to influence the work reported in this paper.

## Data Availability

Data will be made available on request.
